# Pre-exposure to 50 Hz-electromagnetic fields enhanced the antiproliferative efficacy of 5-fluorouracil in breast cancer MCF-7 cells

**DOI:** 10.1371/journal.pone.0192888

**Published:** 2018-04-04

**Authors:** Qi Han, Rui Chen, Fangjie Wang, Sha Chen, Xiongshan Sun, Xiao Guan, Yao Yang, Bingjie Peng, Xiaodong Pan, Jinfang Li, Weijing Yi, Peng Li, Hongwei Zhang, Dongfang Feng, An Chen, Xiaohui Li, Shuhui Li, Zuoming Yin

**Affiliations:** 1 General Hospital of Tibet Area Military Command, Lhasa, China; 2 Department of Clinical Biochemistry, Faculty of Medical Laboratory Science, Southwest Hospital, Third Military Medical University, Chongqing, China; 3 Institute of Materia Medica, College of Pharmacy, Third Military Medical University, Chongqing, China; 4 Tibetan Traditional Medical College, Lhasa, China; 5 Urology, 201th Hospital of People's Liberation Army, Liaoyang, China; Consiglio Nazionale delle Ricerche, ITALY

## Abstract

Resistance to 5-fluorouracil (5-FU) and its induced immune suppression have prevented its extensive application in the clinical treatment of breast cancer. In this study, the combined effect of 50 Hz-EMFs and 5-FU in the treatment of breast cancer was explored. MCF-7 and MCF10A cells were pre-exposed to 50 Hz-EMFs for 0, 2, 4, 8 and 12 h and then treated with different concentrations of 5-FU for 24 h; cell viability was analyzed by MTT assay and flow cytometry. After pre-exposure to 50 Hz-EMFs for 12 h, apoptosis and cell cycle distribution in MCF-7 and MCF10A cells were detected via flow cytometry and DNA synthesis was measured by EdU incorporation assay. Apoptosis-related and cell cycle-related gene and protein expression levels were monitored by qPCR and western blotting. Pre-exposure to 50 Hz-EMFs for 12 h enhanced the antiproliferative effect of 5-FU in breast cancer cell line MCF-7 in a dose-dependent manner but not in normal human breast epithelial cell line MCF10A. Exposure to 50 Hz-EMFs had no effect on apoptosis and P53 expression of MCF-7 and MCF10A cells, whereas it promoted DNA synthesis, induced entry of MCF-7 cells into the S phase of cell cycle, and upregulated the expression levels of cell cycle-related proteins Cyclin D1 and Cyclin E. Considering the pharmacological mechanisms of 5-FU in specifically disrupting DNA synthesis, this enhanced inhibitory effect might have resulted from the specific sensitivity of MCF7 cells in active S phase to 5-FU. Our findings demonstrate the enhanced cytotoxic activity of 5-FU on MCF7 cells through promoting entry into the S phase of the cell cycle via exposure to 50 Hz-EMFs, which provides a novel method of cancer treatment based on the combinatorial use of 50 Hz-EMFs and chemotherapy.

## Introduction

Breast cancer is a deadly disease due to immense difficulties in prevention and treatment[1]. Multidrug resistance of tumor cells is the main reason for the failure of anticancer drugs. Finding novel therapeutic strategies is therefore of great significance in the treatment of highly malignant breast cancer.

5-fluorouracil (5-FU), with the advantages of efficient curative effects and relatively low price, is a broad-spectrum chemotherapeutic drug used to treat a variety of malignancies, including breast cancer and colorectal cancer, as well as cancers of the aerodigestive tract[2]. The mechanism of cytotoxicity of 5-FU has been ascribed to the misincorporation of fluoronucleotides into DNA and inhibit DNA synthesis, thus leading to cell death[2]. However, the lack of tumor specificity and incidence of drug resistance limit the clinical application of 5-FU, resulting in severe side effects and toxicity in the colon and hematologic disorders with immune suppression[3]. Although combination chemotherapy with other compounds such as irinotecan and oxaliplatin has been shown to improve the response rates for advanced colorectal cancer to 40–50% in clinics[4–5], new therapeutic strategies are urgently needed. A substantial amount of evidence has confirmed that extremely low-frequency electromagnetic fields (ELF-EMFs) can have different effects on cell properties. Previous study reported that ELF-EMFs promote cell proliferation in both normal and tumor cells[6], and the possible mechanism is through the action of free radical species[6]. While ELF-EMFs can also inhibit osteosarcoma and other cancer cell growth[7–8], and increased reactive oxygen species (ROS) and p38 MAPK activation may be involved in the mechanism. The influence of ELF-EMFs on properties of breast cancer cells has also drawn wide attention from last centry. The hypothesis that exposure to power frequency (50–60 Hz) magnetic fields increases the risk of breast cancer was put forward in the 1980s[9]. In recent years, a meta-analysis also concluded that ELF-EMFs can increase the risk of human breast cancer[10], while another study showed that the growth of breast cancer cells was significantly decreased by breast cancer-specific modulation frequencies[11]. In addition, electromagnetic fields can also have different influence on drug sensitivities[12–13]. Therefore, we hypothesize that ELF-EMFs with different exposure parameters may influence the biological properties of breast cancer cells and alter the antiproliferative effect of 5-FU.

## Materials and methods

### Cell culture

The human breast cell line MCF7 was obtained from the Cell Bank of the Committee on Type Culture Collection of the Chinese Academy of Sciences (CCTCC). MCF7 cells were cultured in MEM (Gibco, USA) supplemented with 10% fetal bovine serum (Gibco, USA), 1% non-essential amino acids (Sigma-Aldrich, USA) and 10μg/ml insulin (Nanjing, China). The human breast epithelial cell line MCF10A was obtained from Cobioer Biosciences (Nanjing, China), and it was cultured in MEBM supplemented with 10% heat-inactivated fetal bovine serum, 20 ng/ml human epidernal growth factor (EGF), 100 ng/ml cholera toxin, 0.01 mg/ml bovine insulin and 500 ng/ml hydrocortisone (all from Cobioer Biosciences).

### Exposure to 50 Hz-EMF

**Fig 1:**
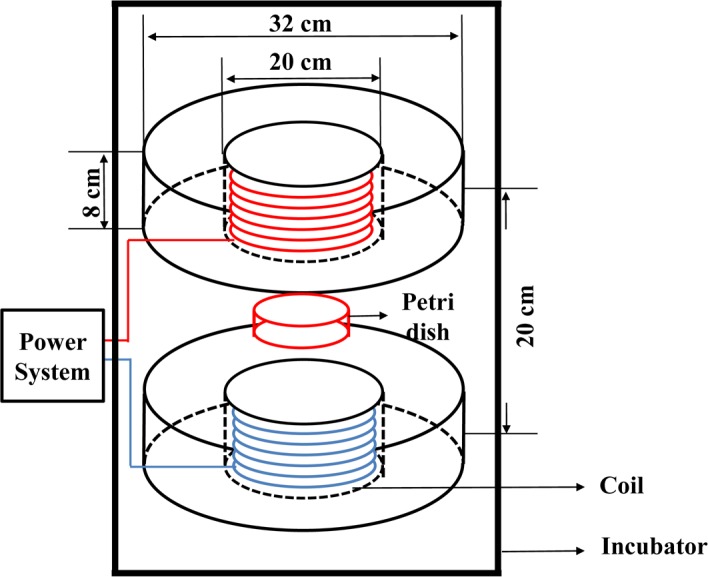
Schematic diagram of the 50 Hz-EMF exposure device.

**Fig 2:**
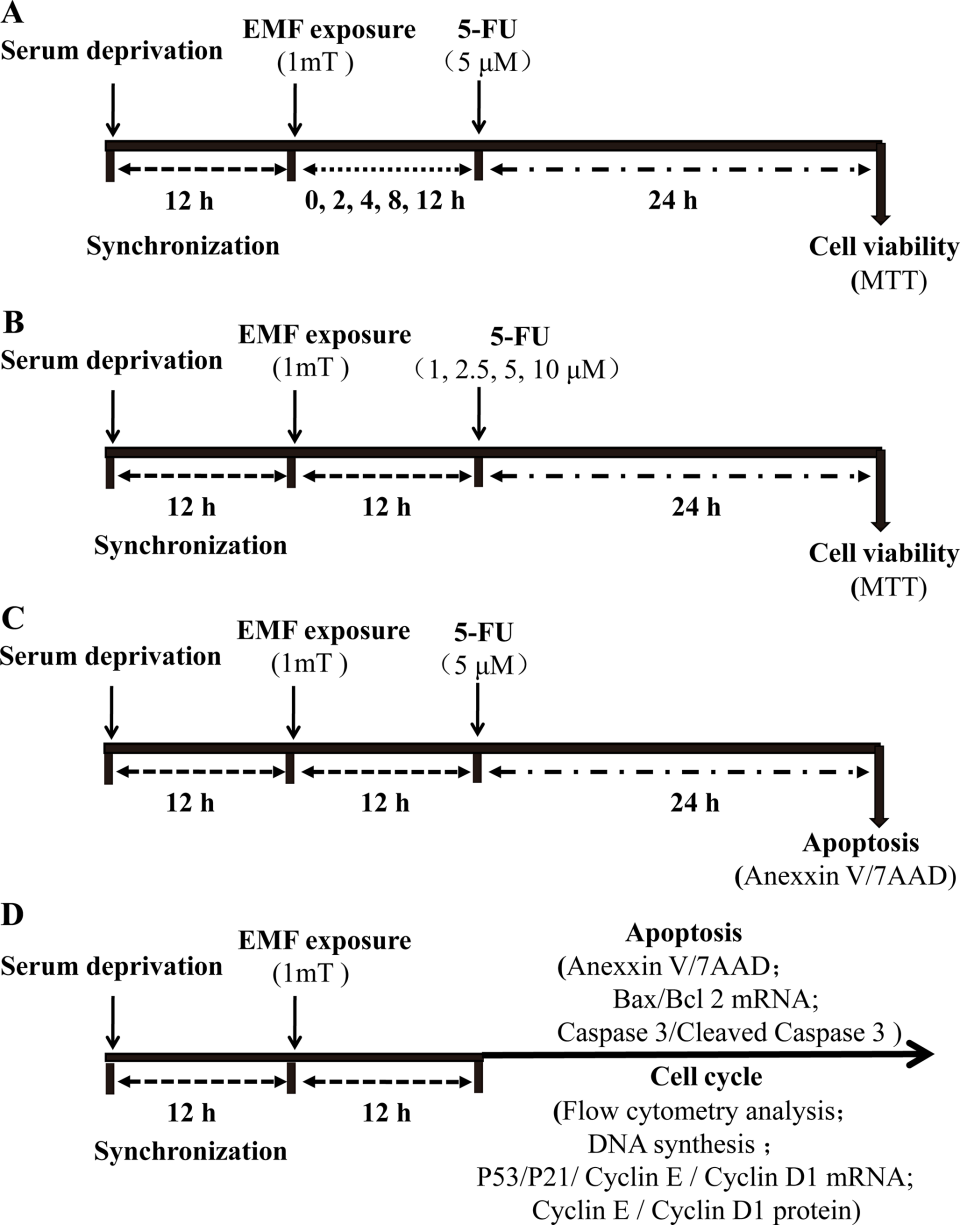
Schematic diagram of the experimental design and exposure time. **(A)** For EMF exposure time assay, MCF7 and MCF10A cells were synchronized by serum deprivation for 12 h, exposed to 50 Hz-EMF (1 mT) for 0, 2, 4, 8 and 12 h, and then the cells were treated with 5-FU (5μM) for 24 h and processed for MTT assay. **(B)** For 5-FU concentration assay, MCF7 and MCF10A cells were synchronized by serum deprivation, exposed to 50 Hz-EMF (1 mT) for 12 h, and then the cells were treated with 5-FU at concentrations of 1, 2.5, 5 and 10 μM for 24 h and processed for MTT assay. **(C)** MCF7 cells were exposed to 50 Hz-EMF (1 mT) for 12 h; then, the cells were treated with 5 μM 5-FU for 24 h, and cell apoptosis was measured by flow cytometry. **(D)** MCF7 and MCF10A cells were synchronized by serum deprivation, exposed to 50 Hz-EMF (1 mT) for 12 h, and then followed by imumunocytochemical and molecular analyses.

The EMF exposure system was constructed according to a previous study[14]. Briefly, the exposure setup mainly consisted of two vertical cylindrical solenoids (8 cm height, 20 cm inner diameter, and 32 cm outer diameter and 850 turns of enameled copper wiring, 1.2 mm diameter, 14 nested layers with 60 turns per layer), which can generate EMFs at amplitudes of 5–1000 μT and frequencies of 1–100 Hz. The solenoid was positioned in a CO_**2**_ incubator to ensure stable environmental conditions (37°C, 5% CO_**2**_ and 95% humidity). In the center of the solenoid as shown in Fig 1, there was a Plexiglas platform for placing cultured cells in Petri dishes. The solenoid was supplied by a power generator, and the frequency and amplitude of EMFs were monitored with a Bartington probe (Bartington Instruments, Oxford, England). Cells in the sham groups were cultured in the same positon of the same incubator in which no magnetic fields existed at the same time as experimental samples and maintained under the same environmental conditions[15]. Two thermometric probes (Homeothermic Control Unit, Harvard Apparatus Ltd., Edenbridge, UK; 0.1°C accuracy) placed in Petri dishes inside and outside the EMF-generating solenoid revealed no marked temperature differences between the culture medium of EMF-exposed and sham cells. After ELF-EMF exposure (50 Hz; 1 mT) the following properties of the cells were evaluated: cell viability after 50 Hz-EMF and 5-FU exposure, cell apoptosis, EdU incorporation, cell cycle distribution, and expression of related mRNA and protein levels (P53, P21, Cyclin E and Cyclin D1) (Fig 2).

### Exposure to 5-FU and cell viability

MCF7 and MCF10A cells in log-phase of growth were divided into two groups,: (i) sham exposure+5-FU group and (ii) 50 Hz-EMF+5-FU group. For EMF exposure time assay, the cells were first synchronized by serum deprivation for 12 h and then exposed to 50 Hz-EMFs at the magnetic intensity of 1 mT for 0, 2, 4, 8 and 12 h. After that, the cells were treated with 5-FU (5 μM) for 24 h. For 5-FU concentration assay, the cells were synchronized by serum deprivation for 12 h, exposed to 50 Hz-EMF (1 mT) for 12 h, and then the cells were treated with 5-FU at concentrations of 1, 2.5, 5 and 10 μM for 24 h. Then cell viability was analyzed using the MTT assay. Briefly, 20 μl of the MTT solution (5 mg/ml in PBS) was added to cells cultured in 96-well plates (1.5×10^4^ cells per well) and incubated at 37°C for 4 h; then, 150 μl of DMSO was added, and OD was detected at 490 nm.

### Monitoring apoptosis by flow cytometric analysis

For cell apoptosis analysis of the combined effect of 50 Hz-EMFs (1 mT, 12 h) and 5-FU (5 μM), cells were harvested after 5-FU treatment for 24 h. For cell apoptosis analysis of the effect of 50 Hz-EMFs, cells were harvested after 50 Hz-EMFs (1 mT) exposure for 12 h. Flow cytometric analysis was conducted according to the manufacturer's instructions. Briefly, 4×10^5^ cells were washed twice with cold PBS and resuspended with 100 μl binding buffer, and then 5 μl of Annexin V-APC and 10 μl of 7AAD were added and incubated at room temperature for 15 min in the dark. After washing two times, cells were resuspended in staining buffer, followed by flow cytometric analysis.

### DNA synthesis analysis

DNA synthesis assay was conducted using a Cell-Light EdU DNA Cell Proliferation Kit (RiboBio, China). Briefly, EdU (20 μM) was added to cells grown on cover slips, and then the cells were exposed to 50 Hz-EMFs at a magnetic intensity of 1 mT for 12 h; then, the cells were fixed with 4% paraformaldehyde, washed and stained with the staining mix for 30 min and counterstained with Hoechst 33342. Finally, the slides were washed with PBS and visualized using a fluorescence microscope (Leica microsystems).

### Cell cycle analysis

Cells were harvested after treatment with 50 Hz-EMFs (1 mT, 12 h), washed with cold PBS and fixed in 75% ice-cold ethanol overnight at 4°C. Then, the fixed cells were stained with 50 μg/ml propidium iodide (PI) containing 50 μg/ml RNase A (DNase free) for 30 min at room temperature in the dark and analyzed with a flow cytometer.

### Western blot analysis

Western blot analysis was performed as described by a previous study[16]. After 50 Hz-EMF exposure (1 mT, 12 h), cells were harvested and lysed in RIPA buffer (Beyotime, China) to obtain protein samples. The protein concentration was determined using a BCA protein assay kit (Beyotime, China). Then, the proteins (30 μg/well) were subjected to 10% SDS-PAGE. After electrophoresis, the proteins were transferred onto a polyvinylidene fluoride membrane (Bio-Rad, USA), blocked and incubated with various primary antibodies at 4°C overnight. Mouse anti-human β-actin (1:1000, Boster, China), rabbit anti-human Cyclin E (1:1000, Santa Cruz, USA), and rabbit anti-human Cyclin D1 were used as primary antibodies. The membranes were washed and incubated with secondary antibodies and then detected by an ECL kit (Millipore, USA).

### Quantitative real-time PCR

Cells were harvested after treatment with 50 Hz-EMFs (1 mT, 12 h), total RNA was extracted by using TRIzol reagent (Invitrogen, USA) according to the manufacturer’s instruction. cDNA was synthesized using Bestar™ qPCR RT Kit (DBI Bioscience, Germany). Quantitative real-time PCR (qRT-PCR) was conducted on a Bio-Rad IQ5 Detection System with Bestar® SYBR Green qPCR Master Mix (DBI Bioscience. The primer sequences are listed in Table 1.

**Table 1:** Primers used for real-time RT-PCR.

Seq name	5'-3' Forward	5'-3' Reverse
P53	GAGGTTGGCTCTGACTGTACC	TCCGTCCCAGTAGATTACCAC
P21	TGTCCGTCAGAACCCATGC	AAAGTCGAAGTTCCATCGCTC
Cyclin D1	GCTGCGAAGTGGAAACCATC	CCTCCTTCTGCACACATTTGAA
Cyclin E	ACTCAACGTGCAAGCCTCG	GCTCAAGAAAGTGCTGATCCC
Bcl-2	GGTGGGGTCATGTGTGTGG	CGGTTCAGGTACTCAGTCATCC
Bax	CAAACTGGTGCTCAAGGCC	GCACTCCCGCCACAAAGAT
β-actin	FCTCCATCCTGGCCTCGCTGT	GCTGTCACCTTCACCGTTCC

### Statistics

All data are expressed as the means ± standard error of the mean (SEM) from three independent experiments performed in duplicate. The data were analyzed by Student’s t-test. The level of significance was set at 0.05.

## Results

### Pre-exposure to 50 Hz-EMFs enhanced the antiproliferative efficacy of 5-FU in breast cancer cell line MCF-7

**Fig 3:**
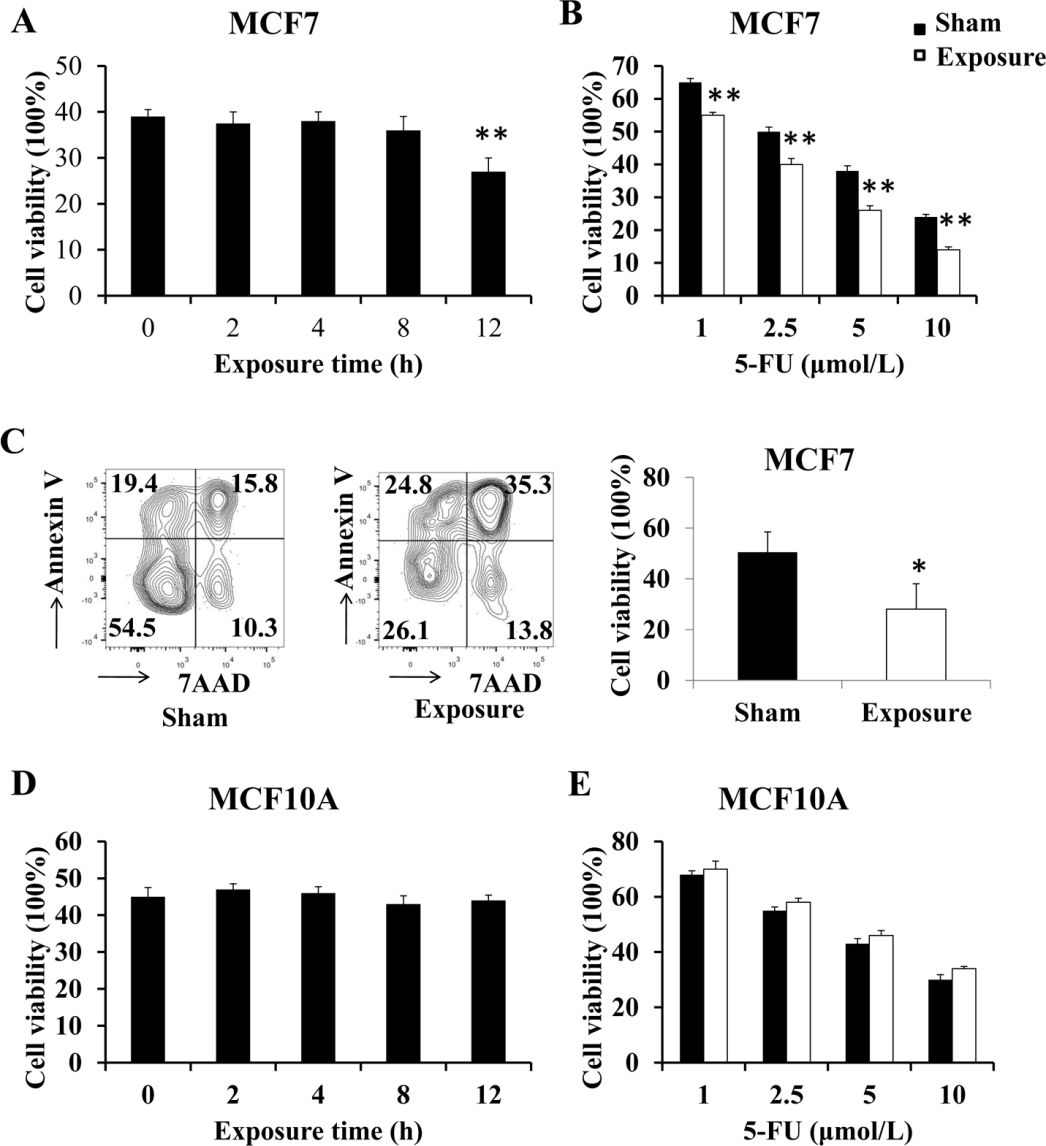
Pre-exposure to 50 Hz-EMFs enhanced the antiproliferative efficacy of 5-FU in breast cancer cell line MCF-7. (A) For EMF exposure time assay, MCF7 cells were exposed to 50 Hz-EMF (1 mT) for 0, 2, 4, 8 and 12 h; then, the cells were treated with 5 μM 5-FU for 24 h, and cell viability was analyzed by the MTT assay. (B) For 5-FU concentration assay, MCF7 cells were exposed to 50 Hz-EMF (1 mT) for 12 h; then, the cells were treated with 1, 2.5, 5 or 10 μM 5-FU for 24 h, and cell viability was analyzed by the MTT assay. (C) MCF7 cells were exposed to 50 Hz-EMF (1 mT) for 12 h; then, the cells were treated with 5 μM 5-FU for 24 h, and cell apoptosis was measured by flow cytometry. (D) and (E) MCF10A cells were subjected to the same treatment as in (A) and (B), respectively. n = 3, *p < 0.05, **p < 0.01.

To analyze the combined effect of 50 Hz-EMFs exposure and 5-FU in MCF7 and MCF10A cells, we first treated cells using a Plexiglas platform (Fig 1). The schematic diagram of the experimental design and exposure time are shown in Fig 2. Briefly, for EMF exposure time assay, MCF7 and MCF10A cells were synchronized by serum deprivation and exposed to 50 Hz-EMFs (1 mT) for 0, 2, 4, 8 and 12 h, and then the cells were treated with 5-FU (5 μM) for 24 h (Fig 2A). Using an MTT-based cell viability assay, we found that exposure to 50 Hz-EMFs for 12 h significantly decreased the survival rate of 5 μM< 5-FU-treated MCF7 cells (Fig 3A). For 5-FU concentration assay, MCF7 and MCF10A cells were synchronized by serum deprivation, exposed to 50 Hz-EMF (1 mT) for 12 h, and then the cells were treated with 5-FU at concentrations of 1, 2.5, 5 and 10 μM for 24 h (Fig 2B). We found that 5-FU in combination with 50 Hz-EMF exposure (1 mT) for 12 h could exhibit better antiproliferative effect on MCF7 cells compared with the 5-FU and sham exposure group, and this is in a dose-dependent manner (Fig 3B). Flow cytometry using Annexin V and 7AAD staining also showed lower viability of 5 μM 5-FU-treated MCF7 cells after 50 Hz-EMF exposure for 12 h (Fig 3C). However, this enhanced antiproliferative effect of 5-FU was not observed in the normal breast epithelial cell line MCF10A after 50 Hz-EMF exposure (Fig 3D and 3E). These results suggested that pre-exposure to 50 Hz-EMF enhanced the therapeutic efficacy of 5-FU in breast cancer cell line MCF-7.

### Exposure to 50 Hz-EMFs alone had no effect on cell apoptosis

**Fig 4:**
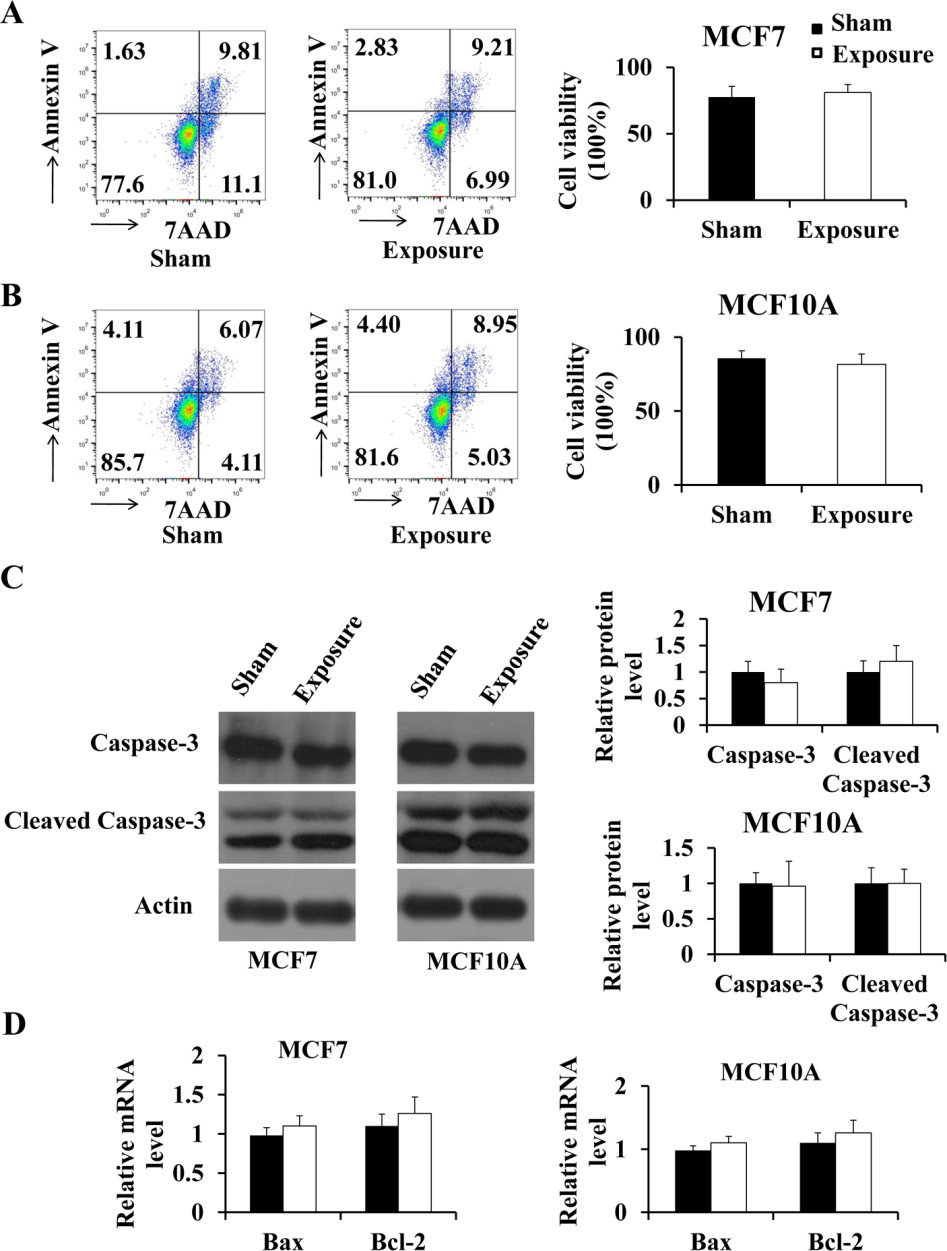
Exposure to 50 Hz-EMFs did not affect apoptosis in MCF7 and MCF10A cells. (A) and (B) Apoptosis in MCF7 and MCF10A cells was measured by flow cytometry after the cells were exposed to 50 Hz-EMFs (1 mT) for 12 h. (C) Left: Caspase-3 and cleaved caspase-3 protein levels were analyzed by western blotting after exposure of MCF7 and MCF10A cells to 50 Hz ELF-EMFs. Right: Quantification of the detected proteins after normalizing to β-actin. (D) Bax and Bcl-2 mRNA expression levels in MCF7 and MCF10A cells were detected by real-time PCR after 50 Hz-EMF exposure. n = 3.

As exposure to 50 Hz-EMFs can improve the cytotoxic effect of 5-FU on MCF7 cells, we initially thought that the exposure to 50 Hz-EMFs alone may induce apoptosis in breast cells. To our surprise, after 50 Hz-EMFs (1 mT, 12 h) exposure (Fig 2C), there were no significant differences in apoptosis between the sham and exposed groups by flow cytometry in MCF7 and MCF10A cells (Fig 4A and 4B). Moreover, we found no alteration in the protein levels of caspase-3 and cleaved caspase-3 in MCF7 and MCF10A cells, two cell apoptosis markers[17], after 50 Hz-EMF exposure by western blot (Fig 4C). Previous studies have shown that EMF exposure may induce transcriptional changes of apoptosis-related genes, such as the pro-apoptotic gene Bax and the anti-apoptotic gene Bcl-2[18]. Thus, we also detected the mRNA expression of Bax and Bcl-2, and no change was found after 50 Hz-EMF exposure in MCF7 and MCF10A cells (Fig 4D). These results suggested that there was no effect of 50 Hz-EMF exposure on cell apoptosis.

### Exposure to 50 Hz-EMFs alone increased DNA synthesis and induced more MCF7 cells to enter the S phase of cell cycle

**Fig 5:**
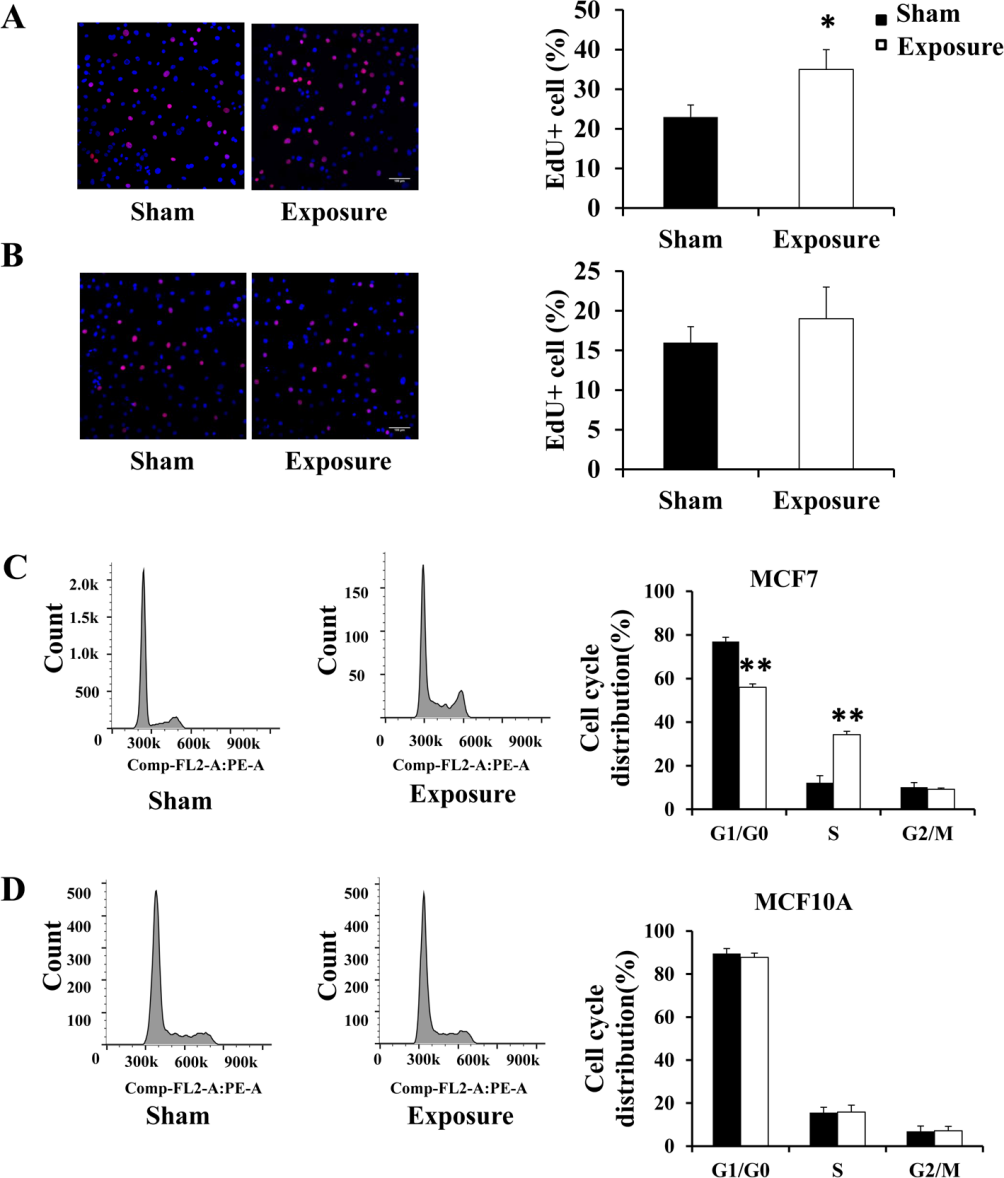
Exposure to 50 Hz-EMFs increased DNA synthesis and induced more MCF7 cells to enter the S phase of cell cycle. (A) and (B) Left: Representative images of EdU/Hoechst 33342 double stained cells (red/blue) after MCF7 and MCF10A cells were exposed to 50 Hz-EMFs (1 mT) for 12 h. Right: The percentage of EdU positive cells was quantified in four random fields in each group. Scale bar: 100 μm. (C) and (D) Cell cycle distribution of MCF7 and MCF10A cells was measured by flow cytometry after the cells were exposed to 50 Hz-EMFs (1 mT) for 12 h. The percentage of cells in each phase was analyzed on the right panel. n = 3, *p < 0.05, **p < 0.01.

Since exposure to 50 Hz-EMFs had no effect on cell apoptosis, and the pharmacological mechanism of 5-FU is specifically targetting DNA synthesis, we then detected the effect of 50 Hz-EMF on DNA synthesis and cell cycle. Using EdU incorporation assay, we observed increased DNA synthesis in the 50 Hz-EMF exposure group compared with the sham group of MCF7 cells (Fig 5A). By flow cytometry, we also confirmed the results that 50 Hz-EMF exposure induced more MCF7 cells to enter the S phase of cell cycle (Fig 5C), while such alterations were not observed in MCF10A cells (Fig 5B and 5D). Given that 5-FU exerts its anticancer effects through the inhibition of DNA synthesis in S phase cells, the increased cytotoxic activity of 5-FU may have resulted from the enhanced sensitivity of MCF7 cells that were in active S phase.

### Exposure to 50 Hz-EMFs upregulated Cyclin E and Cyclin D1 in MCF7 cells

**Fig 6:**
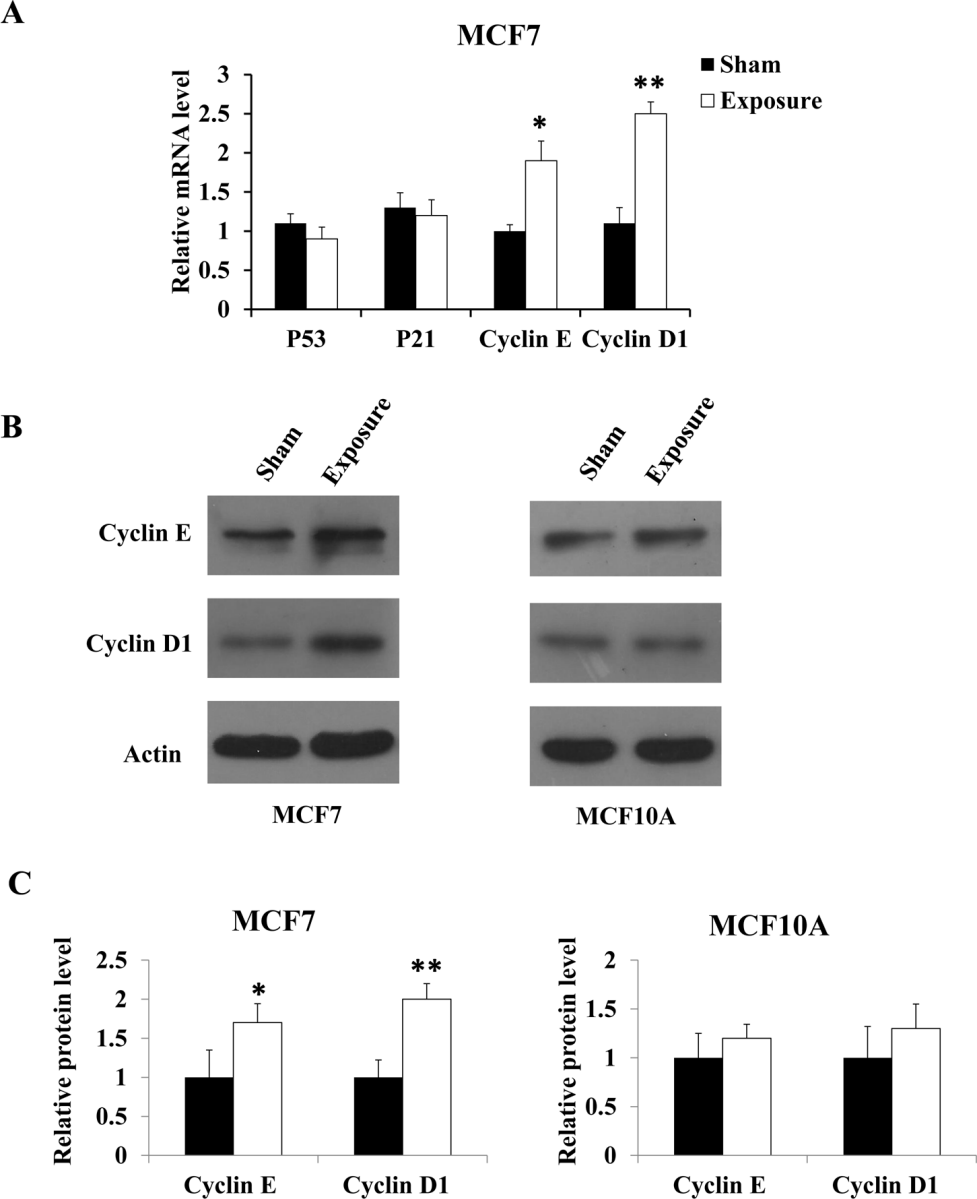
Exposure to 50 Hz-EMFs upregulated Cyclin E and Cyclin D1 expression levels in MCF7 cells. (A) The mRNA expression levels of P53, P21, Cyclin E and Cyclin D1 were measured by qPCR after MCF7 cells were exposed to 50 Hz-EMF (1 mT) for 12 h. (B) The protein expression levels of Cyclin E and Cyclin D1 were measured by western blotting after MCF7 and MCF10A cells were exposed to 50 Hz-EMF (1 mT) for 12 h. (C) Quantification of the detected proteins after normalizing to β-actin. n = 3, *p < 0.05, **p < 0.01.

Since there is a link between exposure to 50 Hz-EMFs and the G1 to S phase transition in MCF7 cells, we sought to identify the cell cycle regulators that may be affected by 50 Hz-EMF exposure. We first examined cell cycle-regulated genes in the S phase, namely, P53, P21, Cyclin E and Cyclin D1. We found no significant differences in the mRNA expression of P53 and P21 in MCF7 cells, the two main genes that play a vital role in the regulation of G1 to S phase transition. Notably, two master regulators of G1 to S phase transition, Cyclin D1 and Cyclin E1[19], were upregulated in the 50 Hz-EMF exposure group compared with the sham group in MCF7 cells (Fig 6A), which was also confirmed by western blot analysis (Fig 6B and 6C). Cyclin E and Cyclin D1 levels were not found to be altered in MCF10A cells after 50 Hz-EMF exposure (Fig 6B and 6C). These results suggested that exposure to 50 Hz-EMFs promoted more MCF7 cells to enter the S phase of cell cycle by upregulating Cyclin E and Cyclin, and because cells in S phase are more sensitive to 5-FU, this lead to an increase in the cytotoxic effect of 5-FU on the cancer cells.

## Discussion

The combination of different antitumor treatment strategies has greatly improved therapeutic efficacy in clinics. For example, combined chemotherapy can not only improve the therapeutic outcome by overcoming multidrug resistance and disrupting multiple cell survival pathways but also improve patient compliance due to reduced dosage of each agent[20–22]. However, the potential effects of combination of electromagnetic fields (EMFs) and chemotherapeutic drugs are usually controversial. It has been shown that stimulation with pulsing electromagnetic fields can enhance the antiproliferative effect of doxorubicin on mouse osteosarcoma cells [12], while other report demonstrated that pre-exposure to electromagnetic fields appear to protect HL-60 cells from the toxic effects of subsequent treatment with doxorubicin [13]. First, the inconsistent effects can be explained by the heterogeneity of various cancer cells. On the other hand, the exposure conditions, such as magnetic intensity, time and frequency, are the key factors influencing the biological effects of EMFs on cancer cells. In this study, we checked the antitumor effect of 5-FU on MCF7 cells with different 50 Hz-EMF pre-exposure durations. We found that the cytotoxic effect of 5-FU was significantly increased by a 12 h pre-exposure time. However, this pre-exposure condition did not enhance the effect of 5-FU on the normal breast epithelial cell line MCF10A. The potential effect of exposure to ELF EMF for human health have been investigated for many years. Several studies showed that ELF EMF can increase incidence of certain types of cancer[23–24]. While some available evidence showed ELF EMF has no effect on cell cycle distribution and apoptosis[25] and cell growth[26]. Furthermore, the anticarcinogenic ability of low-energy electromagnetic waves has also been demonstrated in several *in vitro* studies[11,27–31]. Some studies verified the anticancer effect of EMFs *in vivo*[32–34] or even in patients[35]. These conflicting data might be also due to the differences in frequency, intensity, duration and cell types. However, the effect of 50 Hz-EMF exposure on the physiology of breast cancer MCF7 cells had not been reported. Initially, we also thought that exposure to 50 Hz-EMFs may enhance the antiproliferative effect of 5-FU by inducing cancer cell apoptosis. Surprisingly, we found that 50 Hz-EMF exposure for 12 h had no effect on cell apoptosis but could promote the entry of MCF7 cells into S phase and increase DNA synthesis, thus rendering MCF7 cells in the active phase of S phase more sensitive to the cell cycle-specific drug 5-FU. The mechanism of cytotoxicity of 5-FU is specifically inhibiting DNA synthesis, and 50 Hz-EMF exposure promotes DNA synthesis of MCF7 cells. In our study, 5-FU and ELF-EMF acts in a synergistic manner, this needs to find the right balance point between the ELF-EMF exposure condition and 5-FU concentration. If we use ELF-EMF to induce breast cancer cells into S period, and 5-FU concentration is strong enough to kill cancer cells, the combination of two component would achieve better antiproliferative effects. Crocetti et al. reported low intensity and frequency of pulsed electromagnetic fields selectively impair viability of breast cancer cells[36], and this difference may result from the different intensity and exposure time used in our study.

The cell culture medium composition has major implications for cell properties[37]. In our study, cell culture medium is quite different for MCF7 and MCF10A cells. As for MCF10A cells, the growth factor (EGF) in cell medium has strong effects on cell proliferation. Except for cell type, the enhanced antiproliferative effect of 5-FU in combination with EMF exposure was not observed in the normal breast epithelial cell line MCF10A maybe also because of the promotion effect of MCF10A medium composition on cell proliferation.P53, functioning as an anticancer gene, can activate DNA repair, induce growth arrest and initiate apoptosis. We found no significant changes in the expression of P53 and its downstream P21 in MCF7 cells after 50 Hz-EMF exposure. This was consistent with other studies[38–40]. The cell cycle is tightly regulated by a series of cyclins and cyclin-dependent kinase (CDKs), which are the checkpoints for cell cycle progression at each stage[41]. Dysregulated expression of G1-phase cyclins has been correlated with the initiation of a large proportion of human malignancies[42]. We found significantly increased expression of cell cycle-regulated genes Cyclin E and Cyclin D1 in MCF7 cells after 50 Hz-EMF exposure, which promoted more MCF7 cells to enter the S phase, rendering them more sensitive to 5-FU. This phenomenon was not observed in MCF10A cells, which may be due to differences in cell properties and cell culture medium between normal cells and tumor cells. This has given us great inspiration, in the future, if we can use ELF-EMF to induce cancer cells into specific period of cell cycle, and then use relative cell cycle specific agents to kill cancer cells, which could possibly lead to a cure for cancer.

In conclusion, our study showed that the enhanced antiproliferative effect of 5-FU was caused by the specificity of 5-FU during DNA synthesis due to an increase in Cyclin E and Cyclin D1 expression after exposure to 50 Hz-EMFs, which may provide novel insights for the clinical treatment of breast cancer.
